# Effectiveness of Individualized Physical Exercise in COVID-19–Induced Postural Orthostatic Tachycardia Syndrome

**DOI:** 10.1016/j.jacadv.2026.103328

**Published:** 2026-09-22

**Authors:** Annie M. Svensson, Anna UM. Svensson-Raskh, Anna C. Törnberg, Carl E. Hallberg, Linda Holmström, Artur Fedorowski, Malin C. Nygren-Bonnier

**Affiliations:** a Division of Physiotherapy, Department of Neurobiology, Care Sciences and Society, Karolinska Institutet, Stockholm, Sweden; b Medical Unit Allied Health Professional, Women’s Health and Allied Health Professional Theme, Karolinska University Hospital, Stockholm, Sweden; c Department of Clinical Neuroscience, Karolinska Institutet, Stockholm, Sweden; d Department of Women’s and Children’s Health, Karolinska Institutet, Stockholm, Sweden; e Department of Medicine Solna, Karolinska Institutet, Stockholm, Sweden; f Department of Cardiology, Karolinska University Hospital, Stockholm, Sweden

**Keywords:** clinical research, dysautonomia, physiotherapy, post-COVID-19 condition, randomized controlled trial, rehabilitation

## Abstract

**Background:**

Postural orthostatic tachycardia syndrome (POTS) is common in post-COVID-19 condition and is associated with high symptom burden and reduced quality of life. Although physical exercise is recommended for POTS, evidence of its effectiveness remains limited.

**Objectives:**

The objectives of the study was to evaluate the effectiveness of tailored exercise on health-related quality of life (HRQoL), upright time, physical function, and symptom burden in individuals with COVID-19–induced POTS.

**Methods:**

Adults with COVID-19–induced POTS were randomized to standard care or standard care plus tailored exercise with weekly supervision. Assessments were conducted at baseline and 16 weeks. Primary outcomes were HRQoL and upright time. Secondary outcomes included daily steps, sedentary time, orthostatic autonomic response, muscle strength, physical capacity, patient-reported activity limitations, mental health, and symptom burden. Analyses used mixed models.

**Results:**

Data were analyzed from sixty participants (85% females, mean age 37 years). No time-by-group interaction effects were observed for HRQoL. Upright time was reduced in the intervention group compared with controls, with an estimated between-group difference of −31 min/day, (95% CI -57 to −6). For secondary outcomes, daily steps were reduced in the intervention group, whereas both groups improved in POTS symptoms, activity limitations, and physical capacity, with a trend toward reduced postexertional malaise. No other secondary outcomes showed significant effects.

**Conclusions:**

Tailored exercise did not outperform standard care in HRQoL and was associated with reduced upright time, whereas improvements in several secondary outcomes were observed in both groups. Heterogeneity supports an individualized approach to exercise, integrated with pharmacological and multimodal management strategies.

Postural orthostatic tachycardia syndrome (POTS) is a disorder of the autonomic nervous system characterized by orthostatic intolerance, that is, palpitations and dizziness on standing or while in an upright position, as well as general and less specific dysautonomia symptoms such as fatigue, headache, cognitive impairment, exercise intolerance, sleep disturbances, pain, and gastrointestinal issues.^1^ POTS has received increasing attention in recent years following the outbreak of the COVID-19 pandemic, as a substantial number of individuals have developed POTS after COVID-19.^2^ The syndrome occurs in approximately one-third of those with post-COVID-19 condition (PCC).^3^^,^^4^ POTS has been identified as a phenotype or subcategory of PCC that requires specific treatment, as individuals with PCC-POTS tend to have a higher symptom burden,^3^, ^4^, ^5^ lower health-related quality of life (HRQoL), reduced physical capacity, and higher comorbidity compared with people who have PCC without POTS.^3^^,^^4^ Currently, there is no curative treatment available for either POTS or PCC; however, rehabilitation is recommended to relieve symptoms, improve functional capacity, and enhance overall health.^1^^,^^6^, ^7^, ^8^, ^9^, ^10^ Physical exercise is among the most studied and recommended treatment approaches for both POTS and PCC individually.^6^, ^7^, ^8^, ^9^, ^10^, ^11^ Despite this, the evidence for exercise therapy in POTS remains limited, as most studies are nonrandomized and primarily focus on hemodynamic outcomes.^6^ There is also a wide variation in dropout rates and in how transparently dropout reasons are reported, which may impact the generalizability of previous studies.^6^^,^^12^ The need for individualized exercise interventions has been recognized as an important factor in successful treatment, both for POTS^13^ and PCC,^8^^,^^12^ as fatigue and post-exertional malaise (PEM) are barriers to successful physical training and study participation.^8^^,^^12^, ^13^, ^14^ Overall, regular individualized physical exercise is recommended for individuals with PCC-POTS, but scientific evidence is still sparse to support it as an effective treatment. Therefore, this study aimed to evaluate the effectiveness of an individually tailored exercise intervention compared to standard care on HRQoL, time spent upright, symptom burden, and physical and psychological functioning in individuals with POTS following COVID-19.

## Method

### Design

This was an open-label, randomized controlled parallel-group trial with assessments at baseline and after 16 weeks. The study was approved by the Swedish Ethical Review Authority (2020-02149 with amendments) and registered at ClinicalTrials.gov (NCT05877534). Participants provided written informed consent after receiving oral and written information about the study.

### Participants

Adults (≥18 years) with POTS following COVID-19 or PCC were included. Exclusion criteria were pregnancy, ongoing cancer, cognitive or physical impairments preventing participation, or ongoing tailored exercise. Participants were recruited in Stockholm through Karolinska University Hospital, primary care rehabilitation, and specialized physiotherapy clinics. Eligible individuals identified through referral screening were contacted by telephone and informed about the study.

### Randomization and masking

The randomization sequence was generated by a blinded researcher using block randomization (size 12), with 1:1 allocation and stratified by sex due to the expected low number of male participants. Allocation was concealed in sealed envelopes opened by the test leader after baseline assessment. A different test leader conducted the follow-up assessments and remained blind to group allocation; however, participants and physiotherapists were not blinded due to the nature of the intervention.

### Intervention

Both groups received standard care during the study period (Figure 1). The control group received standard care only and was restricted from POTS-specific or tailored exercise programs for 16 weeks; although alternative activities (eg, walking, breathing exercises, flexibility, or balance training) were permitted. The intervention was offered to the control group after study completion.

**Figure 1:**
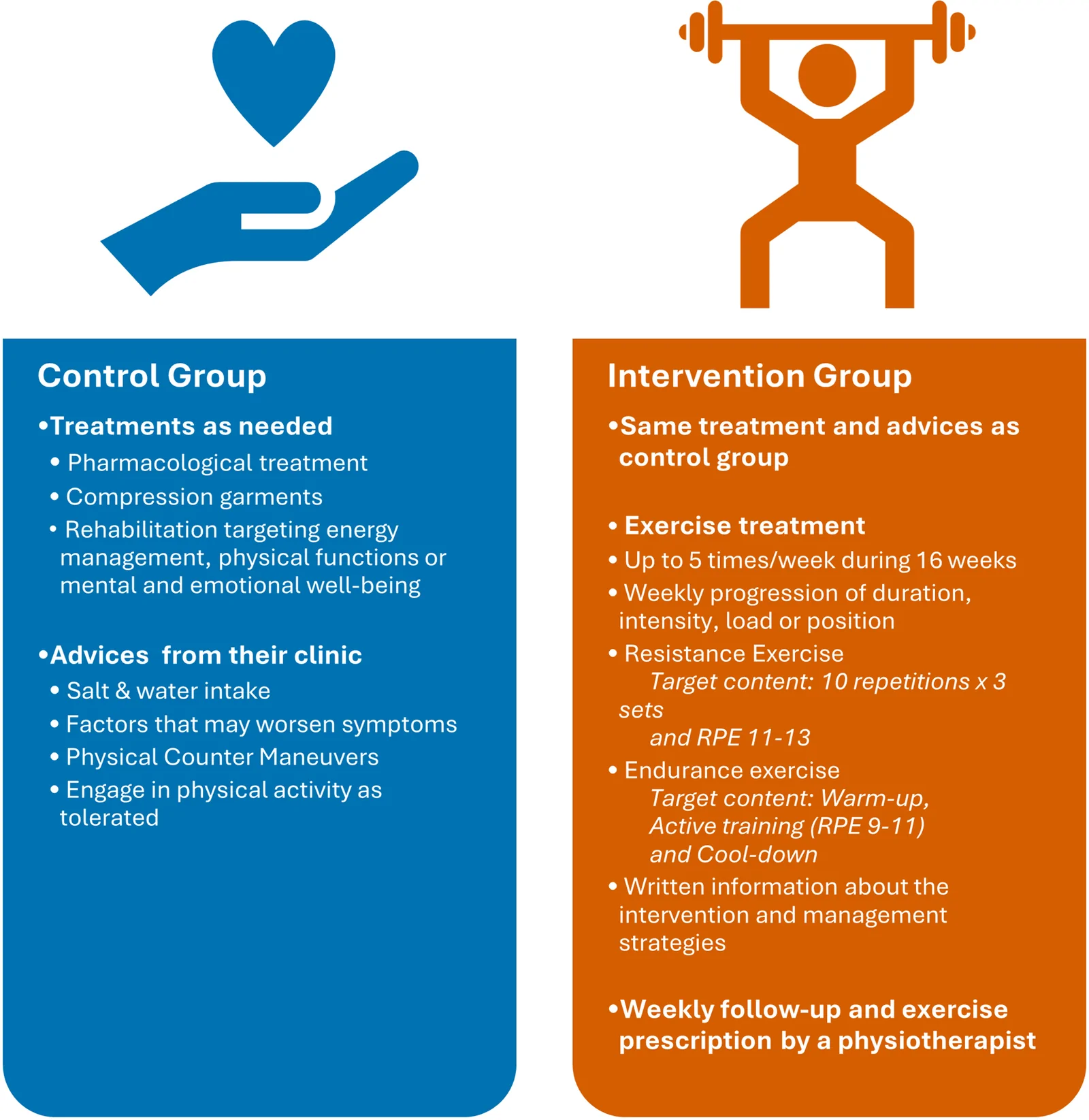
Overview of the Content for the Control and Intervention Group The figure provides an overview of the structure and key components of the control and intervention groups. The control group received standard care alone, and the intervention group received standard care and tailored exercise and weekly supervision in addition. RPE = rating of perceived exertion.

The intervention group performed a 16-week, individually tailored exercise program (up to 5 sessions/week) including endurance and resistance training (Figure 1), with allowance for alternative activities if needed. The intervention was developed based on prior research^6^^,^^10^^,^^15^ and consisted primarily of exercises performed in a supine/semirecumbent position, with personalized guidelines for low-moderate intensity,^16^ dosage, and gradual progression (Figure 1, Supplemental Methods). Progression (eg, increased duration, intensity, load, or position) was paused if symptom exacerbation, including PEM, persisted beyond 24 hours. Participants were supervised weekly by a physiotherapist who reviewed exercise performance, dosage, and symptoms and adjusted and tailored the exercise prescriptions and management strategies accordingly. The training could be conducted at a gym, physiotherapy clinic, or at home, individually or in groups; the setting was adapted to individual needs and could vary throughout the intervention period. Participants in the intervention group were instructed to record daily whether they had performed exercise using an electronic exercise log (or a paper-based exercise log when app use was not feasible), and to rate the perceived intensity of each exercise session.

#### Intervention providers

Physiotherapists affiliated with Region Stockholm’s post-COVID network were invited to participate in the study as health care providers. Participation was voluntary, with no specific inclusion criteria for treatment providers. All participating health care providers received oral and written study information and treatment delivery guidelines and were invited to regular monitoring meetings during the study.

### Data collection

Outcome measures were assessed at baseline and after 16 weeks, following identical procedures. All assessments were conducted while participants continued regular medical treatment. Sessions included a brief interview covering demographic information, treatments, employment, and medical history, followed by the Active Standing Test (AST), assessment of muscle strength, and the six-minute walk test (6MWT). After the assessments, the accelerometer was attached to the participant's thigh, and participants completed questionnaires at home in a calm setting. The accelerometer and the questionnaires were then returned by mail.

#### Primary outcomes

The primary outcomes were changes in HRQoL, measured with the EQ-5D-5L^17^ and time spent in an upright position, measured with an accelerometer.^18^

The EQ-5D-5L assesses 5 dimensions at an index (−0.31 to 1),^19^ with each dimension rated on a five-level scale and includes a visual analog scale (EuroQol visual analog scale [EQ-VAS]; 0-100) for self-rated health, where higher scores on the index and VAS indicate better HRQoL.^17^

Time in upright position (standing and walking) was assessed with a thigh-worn triaxial accelerometer.^18^ Participants wore the accelerometer for 1 week, and a 24-hour protocol was applied.

#### Secondary outcomes

Secondary outcomes were assessed for exploratory reasons and included daily steps, sedentary time, physical and autonomic function, mental health, and symptom burden.

##### Objective outcome measures

Daily steps and sedentary time (awake time spent sitting and lying) were extracted from the accelerometer recordings. Isometric lower-limb muscle strength was assessed using a handheld dynamometer,^20^ with the participant seated at 90° hip and knee flexion, and the dynamometer fixed proximally to the talus.^21^ Three trials were performed for each leg. Autonomic responses to upright position were evaluated using the AST, with heart rate changes measured in accordance with the international STARS guidelines.^22^ Submaximal physical capacity was assessed using the 6MWT with standardized instructions.^23^

##### Patient-reported outcome measures

Patient-identified activity limitations were measured with the Patient-Specific Functional Scale (PSFS) (0-10),^24^ in which participants self-reported and rated up to 3 activities affected by their condition. The General Anxiety Disorder 7-item scale (0-28)^25^ and the Patient Health Questionnaire (0-27)^26^ were used to assess anxiety and depression. Symptom burden was assessed by the Fatigue Severity Scale (9-63),^27^ Mental Fatigue Scale (0-42),^28^ DePaul Symptom Questionnaire-PEM (DSQ-PEM) (yes/no),^29^ Insomnia Severity Index (0-28),^30^ Vanderbilt Orthostatic Symptom Score (0-90)^31^ (conducted immediately after AST), and the Malmö POTS Score (0-120).^32^ For all questionnaires except the PSFS and DSQ-PEM, item responses are summed to a total score, with higher scores indicating greater symptom severity or symptom burden. For the PSFS, higher scores indicate less activity limitations, and the DSQ-PEM defines PEM as present when a score ≥2 is reported for both severity and frequency on at least 1 item.

### Statistical methods

Data were collected and managed using a secure, web-based data capture system^33^ hosted at Karolinska Institutet, and analyzed according to the prepublished statistical analysis plan (NCT05877534), including a prespecified modified intention-to-treat approach and power and sample size calculations based on EQ-VAS.

#### Data processing

Data processing was performed before the statistical analysis. For the PSFS, a participant-level mean score was calculated across all activities. Muscle strength values were averaged per limb. Both muscle strength and 6MWT were also expressed as a percentage of predicted values.^21^^,^^34^ For the AST, the heart rate response was calculated as the difference between supine and standing heart rate. Accelerometer data were processed using the manufacturer's software,^18^ with the CREA algorithm applied to classify event-based data, which were aggregated into participant-level daily summaries and observations with ≥4 valid days (wear time ≥14 hours)^35^ were retained in the analysis. Upright time (software-defined standing and stepping time) and sedentary time during waking hours (software-defined time spent sitting and lying during wake hours) were analyzed and expressed in minutes or hours/day. For other variables, either total scores or raw scale values were used in the analysis, as appropriate.

#### Statistical analysis

Baseline demographic and clinical characteristics were summarized descriptively as means with SDs or counts with percentages. Longitudinal changes in continuous outcomes were analyzed using linear mixed models (LMMs) with a random intercept for each participant. Fixed effects included time (baseline vs 16 weeks), group allocation (intervention vs control) and their interaction, with sex as a covariate. Model assumptions were assessed through visual inspection and review of the random-effects structure; mild deviations from normality were considered acceptable given the robustness of mixed models. Due to a highly skewed distribution, analysis of PEM prevalence deviated from the statistical analysis plan, and a Bayesian generalized LMM was applied. To further assess treatment response, an exploratory analysis was performed on participants achieving a clinically meaningful improvement in EQ-VAS (>10 points), and predictors were explored using univariate logistic regression analyses of demographic and baseline characteristics. A multivariable logistic model was subsequently fitted, adjusting for randomization and baseline EQ-VAS, with additional predictors selected based on univariate screening (*P* < 0.200) or clinical relevance. All analyses were conducted in R, version 4.4.3 (R Foundation for Statistical Computing)^36^ using 2-sided tests with α = 0.05. Given the exploratory nature of secondary outcomes and responder analyses, *P* values were interpreted cautiously without multiple testing correction.

### Data sharing statement

Individual-level data are not publicly available as the data are subject to Swedish legislation and the General Data Protection Regulation. Aggregated data are available through the KI Data Repository on reasonable request. Requests for access to the data can be put to the Research Data Office (rdo@ki.se) at Karolinska Institutet and will be reviewed and handled according to relevant legislation and data sharing agreement with the recipient of the data. The study protocol and statistical analysis plan are publicly available at ClinicalTrials.gov, as described previously.

## Results

Participants were recruited from May 2023 to May 2025. Of the 193 individuals assessed for eligibility, 60 were enrolled and allocated to either the intervention or control group. Two participants withdrew during the intervention period (both from the control group), and an additional 4 participants did not complete the 16-week follow-up assessment (Figure 2). Only 1 participant assigned to the intervention group formally discontinued the intervention, for reasons unrelated to the intervention itself. Among participants who remained in the intervention arm, reported exercise exposure varied considerably, ranging from 3 to 72 exercise sessions during the intervention period. The exercise logs showed that 26 of the participants reported at least 1 exercise session, and the median exercise frequency was 2.6 sessions per week, corresponding to 41 sessions per participant over the 16-week intervention period. The mean self-reported exercise intensity was 12.5 (SD 2.0).

**Figure 2:**
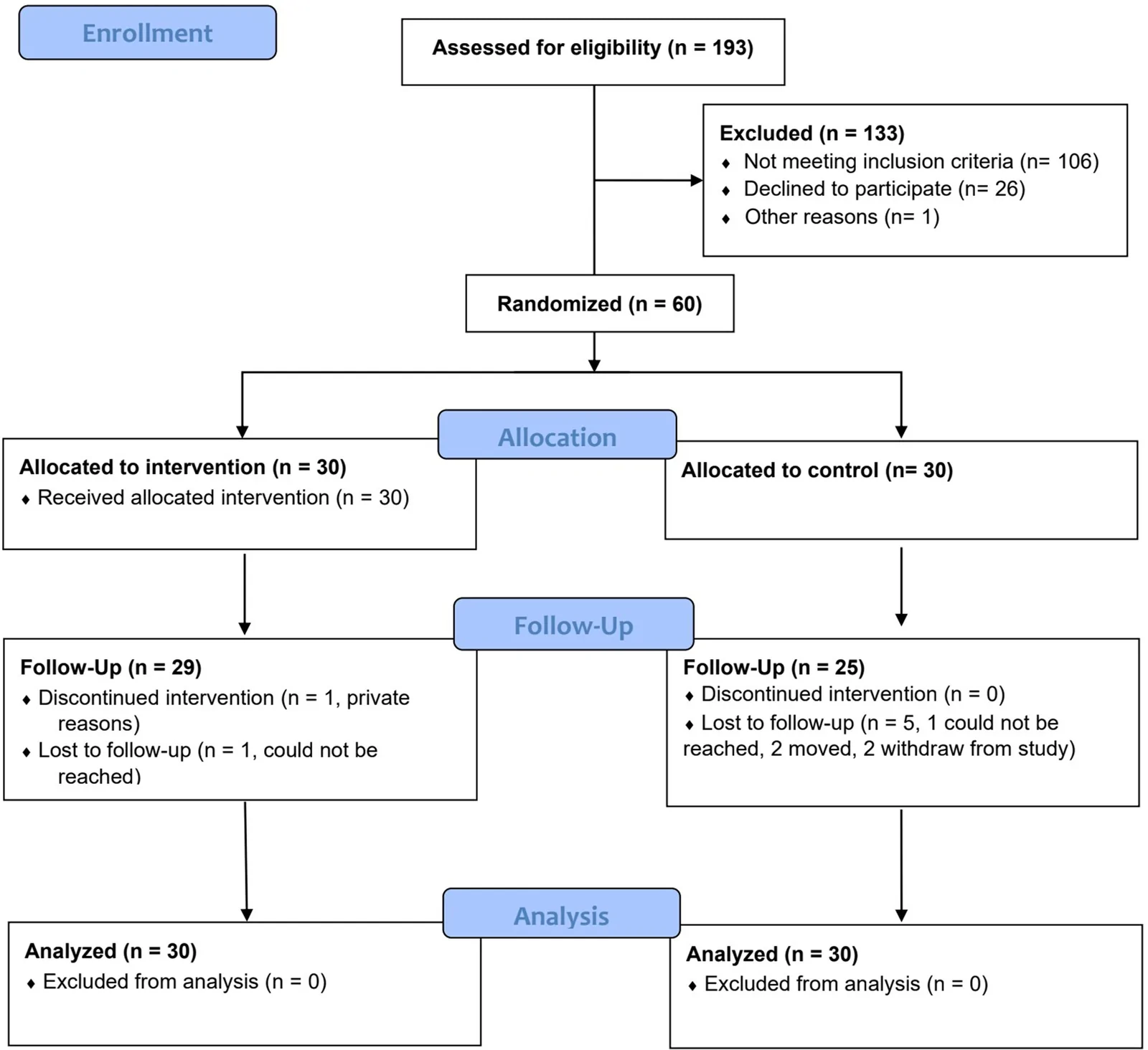
Participant Flowchart This figure illustrates the flow of participants through the study, from initial screening to final analysis. The chart outlines the number of individuals assessed for eligibility, excluded, allocated to the control and intervention groups, and those who completed follow-up and were included in the main analysis.

At baseline, the 2 groups were similar in demographic characteristics and the outcome variables. Notably, the cohort was highly inactive and sedentary, reported low self-rated health, and patient-reported outcome measures (PROMs) revealed a high symptom burden. Muscle strength of both legs was, on average, close to the predicted values, with substantial variability, in line with several of the other outcomes. The prevalence of PEM was very high in both groups, with all participants in the intervention group and 96% in the control group meeting the DSQ-PEM criteria (Tables 1 and 2).

**Table 1:** Participant Characteristics at Baseline

	Control (n = 30)	Intervention (n = 30)
Age (y)	36.4 (10.4)	37.6 (13.3)
BMI (kg/m^2^)	25.2 (5.9)^a^	24.6 (5.7)
Female	86.7% (26)	83.3% (25)
Number of current or previous comorbidities	2.6 (1.6)	3.0 (2.5)
Months since COVID-19 infection	31.3 (11.1)^a^	31.6 (14.1)
Months since POTS diagnosis (median, IQR)	3.5 (3.9)	2.7 (4.9)^a^
Civil status		
Single	41.4% (12)	43.3% (13)
Married	20.7% (6)	26.7% (8)
Cohabitant	24.1% (7)	23.3% (7)
Other	13.8% (4)	6.7% (2)
Missing	1	0
Education		
Elementary school	3.3% (1)	0% (0)
High school	40.0% (12)	30.0% (9)
University	56.7% (17)	70.0% (21)
On sick leave	53.3% (16)	50.0% (15)
Tobacco use		
Current tobacco user	6.7% (2)	23.3% (7)
Previous tobacco user	10.0% (3)	13.3% (4)
Never used tobacco	83.3% (25)	63.3% (19)
Using pharmacological treatment	90.0% (27)	96.7% (29)
Using compression garment	32.1% (9)^b^	32.1% (9)^b^

Data are presented as mean ± SD or as % (n) unless otherwise specified.

BMI = body mass index; POTS = postural orthostatic tachycardia syndrome.

a n = 29.

b n = 28.

**Table 2:** Outcome Measures at Baseline and Follow-Up

	Baseline		Follow-Up	
	Control (n = 30)	Intervention (n = 30)	Control (n = 25)	Intervention (n = 29)
Health-related quality of life				
EQ-VAS	36.7 (18.7)^b^	35.0 (15.7)	44.2 (18.3)^e^	40.9 (15.6)^a^
EQ-5D Index	0.6 (0.1)^c^	0.6 (0.1)	0.7 (0.1)^e^	0.7 (0.1)^a^
Physical activity				
Time in upright position/day (hours)	4.5 (2.3)^d^	4.3 (2.1)^b^	4.4 (1.7)^g^	3.9 (1.8)^c^
Steps/day	6,303 (3,160)^d^	5,745 (3,063)^b^	6,621 (3,241)^g^	4,914 (2,120)^c^
Sedentary time/day (hours)	10.0 (1.5)^d^	10.3 (2.2)^b^	9.44 (1.5)^g^	10.6 (1.6)^c^
Muscle strength				
Right leg (% of predicted)	95.2 (28.9)	86.4 (25.8)	96.1 (24.3)^g^	94.7 (33.3)^b^
Left leg (% of predicted)	98.2 (24.9)^a^	92.1 (26.3)	100.0 (21.4)^g^	97.5 (32.1)^b^
Orthostatic autonomic response				
Δ pulse during AST	20.3 (13.0)	22.8 (12.4)^b^	19.7 (8.8)^g^	25.1 (15.2)^d^
Physical capacity *(% of predicted distance in 6MWT)*	74.1 (13.6)^a^	70.2 (22.2)	79.7 (13.4)^g^	74.3 (22.8)^a^
Patient-reported outcomes				
Patient-identified activity limitations (PSFS_mean_ score)	2.2 (1.4)^c^	1.9 (1.4)	3.1 (1.7)^g^	2.8 (1.9)^b^
Anxiety (total score GAD-7)	7.3 (5.8)^b^	7.8 (6.0)^a^	6.4 (4.5)^f^	7.1 (5.5)^a^
Depression (total score PHQ-9)	12.3 (5.0)^c^	11.2 (5.8)	11.6 (4.9)^e^	11.2 (5.2)^a^
Fatigue (total score FSS)	56.6 (7.0)^b^	55.8 (8.3)	54.7 (8.7)^f^	55.1 (8.5)^c^
Mental fatigue (total score MFS)	21.1 (5.6)^c^	21.0 (6.4)^a^	21.1 (5.5)^g^	20.1 (5.7)^b^
Postexertional malaise	96.4% (27)^b^	100.0% (30)	92.0% (23)^e^	89.7% (26)^a^
Insomnia (total score ISI)	14.9 (5.1)^h^	14.2 (5.8)^d^	15.0 (4.3)^i^	12.6 (6.2)^e^
Orthostatic symptoms (total score VOSS)	28.3 (14.7)	35.6 (19.1)	27.5 (18.0)^f^	36.4 (17.3)^a^
POTS symptoms (total score MaPS)	60.3 (21.2)^b^	62.2 (18.8)^b^	54.0 (24.5)^g^	57.3 (17.7)^a^

Values are mean ± SD unless otherwise specified.

AST = Active Standing Test; EQ-VAS = EuroQol visual analog scale; FSS = Fatigue Severity Scale; GAD-7 = General Anxiety Disorder 7-item; ISI = Insomnia Severity Index; MaPS = Malmö POTS Score; MFS = Mental Fatigue Scale; PHQ-9 = Patient Health Questionnaire; PSFS = Patient-Specific Functional Scale; VOSS = Vanderbilt Orthostatic Symptom Scale; 6MWT = six-minute walk test; other abbreviation as in Table 1.

a n = 29.

b n = 28.

c n = 27.

d n = 26.

e n = 25.

f n = 24.

g n = 23.

h n = 22.

i n = 21.

### Primary outcomes

The primary HRQoL outcomes demonstrated no time-by-group interaction. The EQ-5D index remained stable over time, with no between-group differences (Table 3). Self-rated health (EQ-VAS) improved over time, with no difference between groups (*P* > 0.10), (Table 3, Supplemental Table 1). The LMM revealed substantial between- and within-individual variability (Figure 3). The exploratory responder analysis of EQ-VAS showed that 36% of participants in each group improved ≥10 points. In the univariate analyses, only baseline EQ-VAS and insomnia severity had *P* values <0.200; body mass index and walking distance were additionally included in the multivariate model based on clinical relevance and *P* values <0.250. In the final model, only a lower baseline EQ-VAS was associated with increased likelihood of achieving a clinical meaningful improvement (≥10 points) (Table 4).

**Table 3:** Results From Fixed Effects in Linear Mixed Models

	Outcome	Estimate Time Effect (95% CI)	*P* Value	Estimate Interaction Effect (95% CI)	*P* Value	Estimate Sex Effect (95% CI) *(Covariate)*	*P* Value
Health-related quality of life	EQ-VAS	7.155 (0.5-3.8)	**0.035**	−1.725 (−10.8 to 7.3)	0.704	−1.468 (−13.2 to 10.3)	0.803
	EQ-5D Index	0.034 (0.0 to 0.1)	0.160	−0.004 (−0.1 to 0.1)	0.899	−0.055 (−0.1 to 0.0)	0.246
Daily steps and posture	Upright time (hours/day)	0.143 (−0.2 to 0.5)	0.376	−0.522 (−0.9 to −01)	**0.016**	1.101 (0.4-2.6)	0.148
	Sedentary time (hours/day)	−0.357 (−0.8 to 0.1)	0.128	0.532 (0.1-1.1)	0.091	−1.203 (−2.5 to 0.1)	0.080
	Steps/day	339.606 (−290 to 880)	0.253	−1.151 (−1891 to −323)	**0.004**	1.199 (−1,005 to 3,413)	0.289
Muscle strength	Left leg (N)	3.384 (−22.6 to 29.4)	0.795	10.271 (−25.4 to 45.9)	0.566	−134.166 (−182.1 to −86.2)	**0.000**
	Left leg (% of predicted)	2.176 (−7.7 to 12.0)	0.659	3.296 (−10.1 to 16.7)	0.623	−10.977 (−28.6 to 6.6)	0.216
	Right leg (N)	3.371 (−23.5 to 30.2)	0.802	19.468 (−17.3 to 56.3)	0.293	−138.735 (189.9 to −87.6)	**0.000**
	Right leg (% of predicted)	0.999 (−9.2 to 11.2)	0.846	7.191 (−6.7 to 21.1)	0.304	−13.699 (−32.6 to 5.2)	0.151
Orthostatic autonomic response	Δ pulse during AST	−0.416 (−4.3 to 8.4)	0.870	2.676 (−10.0 to 7.6)	0.452	−6.060 (−14.1 to 2.0)	0.138
Physical capacity (% of predicted distance in 6MWT)		4.638 (0.9-8.4)	**0.016**	−0.378 (−5.4 to 4.6)	0.880	7.422 (−5.6 to 20.4)	0.258
Patient-reported outcomes	Patient-identified activity limitations (PSFS_mean_ score)	0.872 (0.4-1.4)	**0.001**	0.030 (−0.6 to 0.7)	0.927	−1.007 (−2.2 to 0.2)	0.092
	Depression (total score PHQ-9)	−0.795 (−2.3 to 0.7)	0.298	1.116 (−0.9 to 3.2)	0.282	−0.606 (−4.5 to 3.2)	0.754
	Anxiety (total score GAD-7)	−0.868 (−2.4 to 0.6)	0.246	0.440 (−1.6 to 2.5)	0.662	0.707 (−3.4 to 4.8)	0.729
	Fatigue (total score FSS)	−2.397 (−5.3 to 0.5)	0.105	1.598 (−2.4 to 5.6)	0.428	−1.496 (−7.6 to 4.6)	0.624
	Mental fatigue (total score MFS)	−0.993 (−2.5 to 0.5)	0.188	0.307 (1.7-2.3)	0.761	2.422 (−1.9 to 6.7)	0.265
	Insomnia (total score ISI)	−0.344 (−2.4 to 1.7)	0.735	−1.239 (−4.0 to 1.6)	0.376	2.907 (−1.2 to 7.0)	0.164
	Orthostatic symptoms (total VOSS score)	−0.664 (−5.7 to 4.3)	0.791	2.027 (−4.8 to 8.8)	0.551	6.402 (−5.3 to 18.1)	0.279
	POTS symptoms (total score MaPS)	−5.470 (−10.9 to 0.0)	**0.050**	0.972 (−6.4 to 8.4)	0.793	13.010 (−1.8 to 27.9)	0.085

**Bold** typeface was used to denote statistically significant results (*P* < 0.05).

Abbreviations as in Tables 1 and 2.

**Figure 3:**
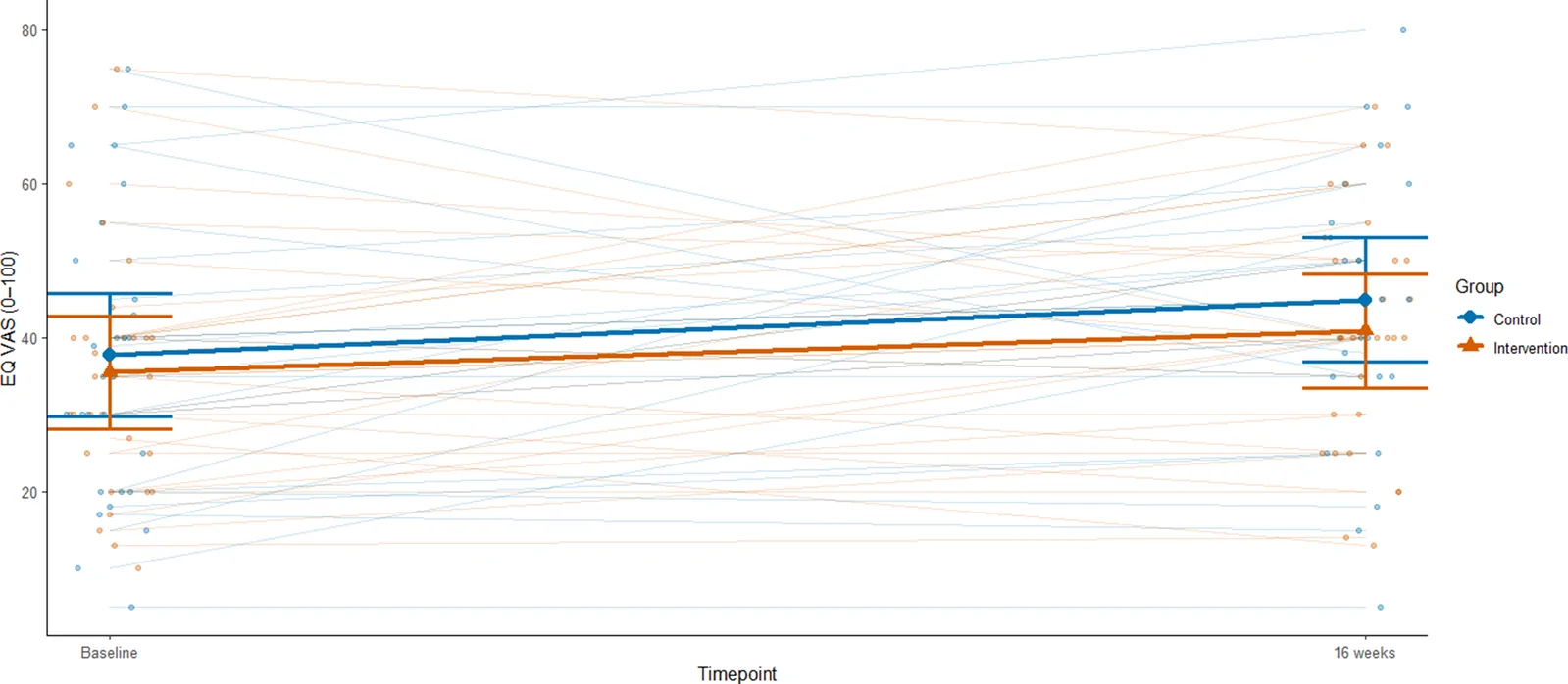
Individual and Model-Estimated EuroQol Visual Analog Scale Trajectories The figure illustrates longitudinal changes in EQ-VAS estimated from the linear mixed model and within-participant changes over time. The plot combines observed individual EQ-VAS measurements (dots) and individual trajectories for participants with assessments at both time points (thin lines). The solid group-specific lines represent the estimated marginal means from the linear mixed model, with error bars indicating 95% CIs. EQ-VAS = EuroQol visual analog scale.

**Table 4:** Logistic Regression to Explore Predictors of Clinically Meaningful Improvement in EQ-VAS

Predictor	OR (95% CI)	*P* Value
Allocated to the intervention group	0.33 (0.06-1.43)	0.155
EQ-VAS at baseline	0.95 (0.89-0.99)	**0.041**
Insomnia severity (total score ISI)	0.87 (0.74-1.00)	0.063
BMI	1.12 (0.98-1.31)	0.131
Physical Capacity (meters covered at 6MWT)	1.00 (0.99-1.00)	0.490

Clinically meaningful improvement = ≥10 U. **Bold** typeface was used to denote statistically significant results (*P* < 0.05).

Abbreviations as in Tables 1 and 2.

For the primary outcome of upright time, the LMM revealed an interaction between group allocation and time (*P* < 0.05) (Table 3, Figure 4), corresponding to a 31-minute greater reduction in upright time/day in the intervention group compared to the control group (Figure 4, Supplemental Table 2). Upright time decreased from baseline to 16 weeks in the intervention group, whereas no change was observed for the control group (Supplemental Table 2).

**Figure 4:**
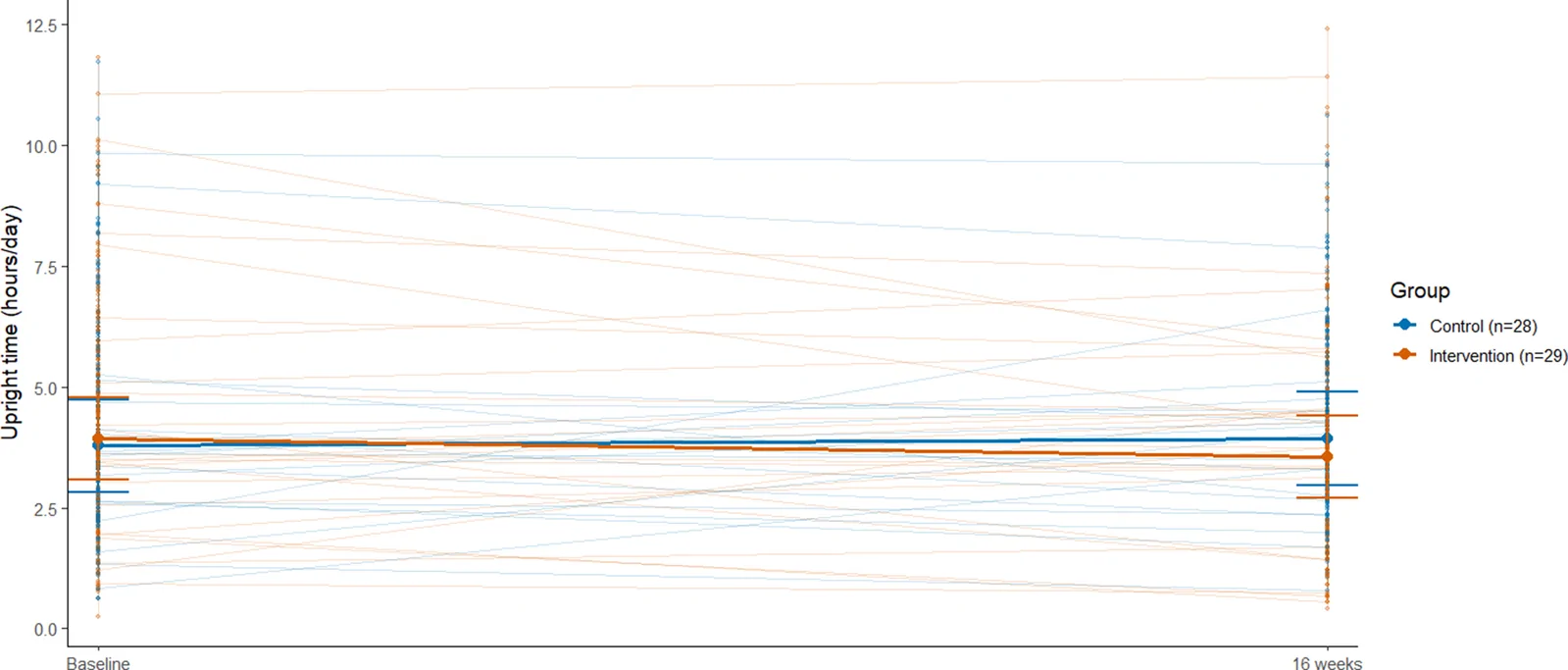
Individual and Model-Estimated Upright Time The figure illustrates longitudinal change in upright time (hours/day) estimated from the linear mixed model and within-participant changes over time. The plot combines observed individual measurements (dots) and individual trajectories for participants with assessments at both time points (thin lines). The solid group-specific lines represent estimated marginal means from the linear mixed model, and error bars indicate 95% CIs.

### Secondary outcomes

Several secondary outcomes improved over the 16 weeks (Table 2). POTS symptoms (Malmö POTS Score) showed a reduction over time, without a time-by-group interaction (*P* > 0.10) (Table 3, Supplemental Table 3). Patient-identified activity limitations (PSFS) and physical capacity (% predicted distance at 6MWT) improved in both groups, but without interaction effects (*P* > 0.1), indicating comparable gains (Table 3, Supplemental Tables 4 and 5). In contrast, an interaction was observed for daily steps, with a greater reduction in the intervention group (*P* < 0.01) (Table 3). Importantly, the high prevalence of PEM declined over time in both groups (Table 2, Figure 5), with a tendency toward a larger reduction in the intervention group, although uncertainty was substantial (OR: 0.61; 95% credible interval: 0.13-2.75). For the remaining secondary outcomes, no significant changes over time or between-group differences were observed, and sex had no effect in any model; although females had lower absolute muscle strength than males, no sex differences were observed in the predicted values (Table 3). The overall pattern of findings is illustrated in the Central Illustration.

**Figure 5:**
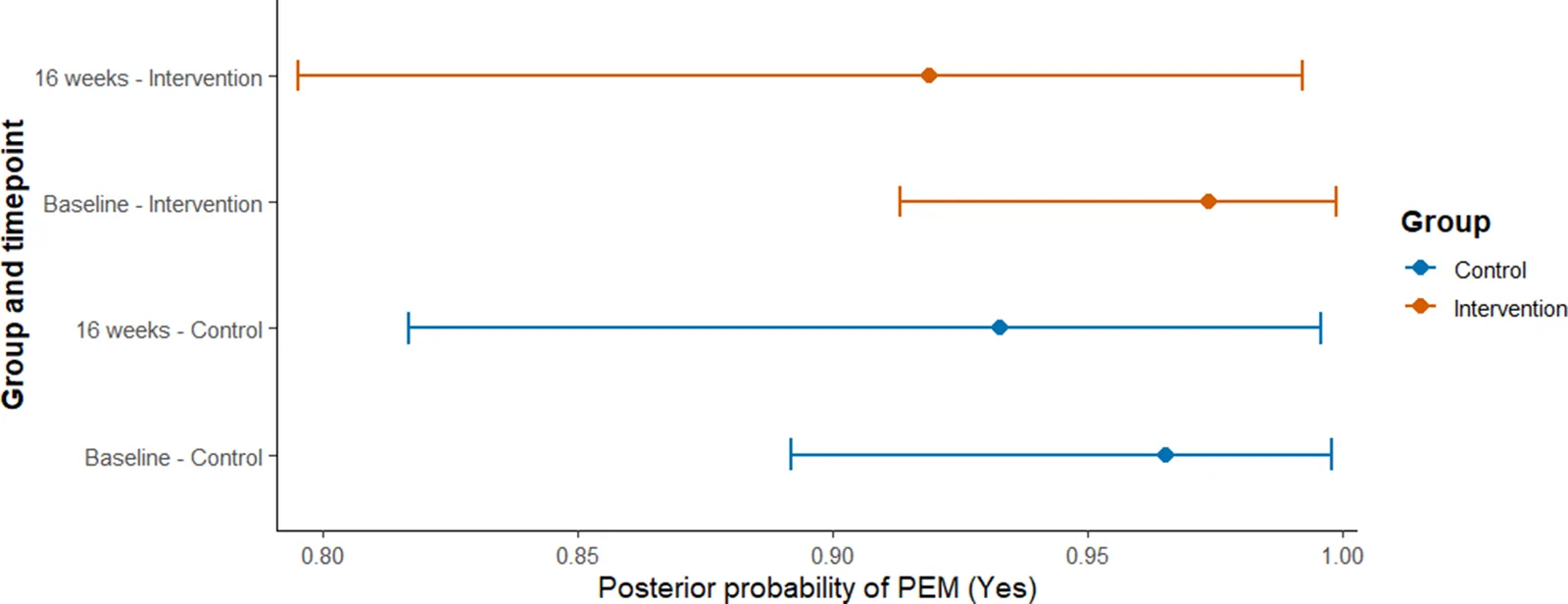
Posterior Probabilities of Postexertional Malaise The figure illustrates model-estimated posterior probabilities of postexertional malaise (PEM) by group and time point. Points represent posterior mean probabilities, and horizontal bars represent 95% Bayesian credible intervals derived from a Bayesian generalized linear mixed model adjusting for sex.

**Central Illustration:**
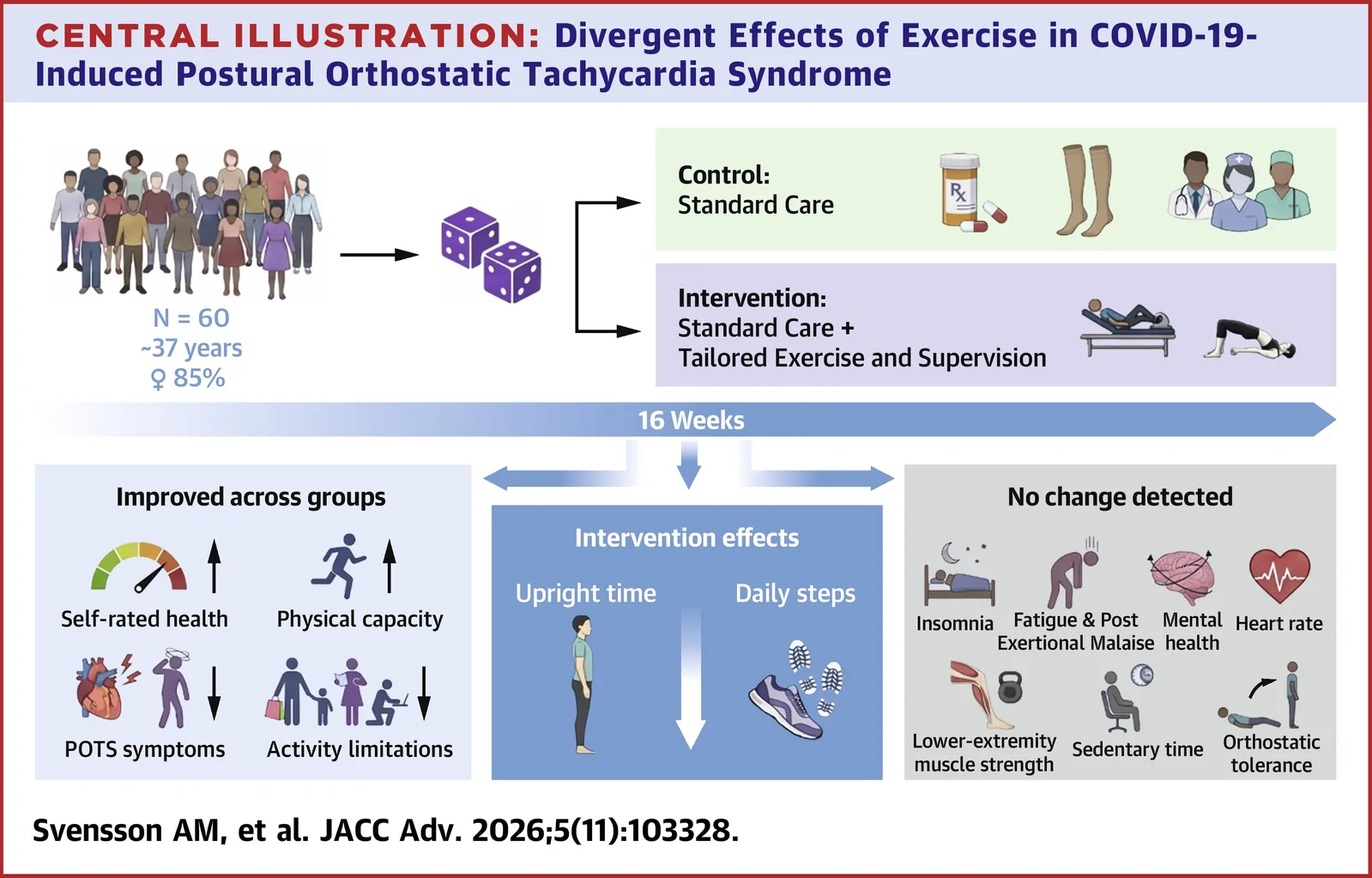
Divergent Effects of Exercise in COVID-19-Induced Postural Orthostatic Tachycardia Syndrome Among 60 participants with COVID-induced POTS, a 16-week program of tailored exercise with weekly supervision was compared with standard care alone. Exercise was associated with reduced upright time and daily steps, whereas self-rated health, physical capacity, POTS symptoms, and activity limitations improved in both groups. Several outcomes remained unchanged. POTS = postural orthostatic tachycardia syndrome.

## Discussion

In this study, we evaluated tailored and supervised exercise intervention in individuals with COVID-19–induced POTS, examining effects on HRQoL, upright time, and secondary outcomes including symptom burden, physical capacity, and orthostatic tolerance, compared with standard care. Overall, no between-group differences were observed in HRQoL, although upright time decreased significantly in the intervention group. Secondary exploratory outcomes, including POTS symptoms, physical capacity, and patient-identified activity limitations, improved over time. These findings suggest that the intervention did not confer additional benefits beyond standard care. Standard care in POTS is multimodal^1^^,^^5^ and such comprehensive care may itself lead to meaningful improvements, limiting the ability to detect exercise-specific effects. Moreover, standard care may have evolved since the prepandemic period due to the increased POTS prevalence,^2^ possibly with increased awareness and access to care^5^ compared to prepandemic studies.

The findings should be interpreted in the light of the limited and methodologically heterogeneous scientific evidence for exercise interventions in POTS, as to date, only 1 previous randomized controlled trial has evaluated the effects of physical exercise^6^^,^^37^ and reported improvements in selected HRQoL subdomains^37^ that are not directly comparable to those assessed with EQ-5D-5L. Another recent feasibility trial by McGregor et al^38^ included EQ-5D as a secondary outcome and similarly reported trends toward improvement. By assessing HRQoL as a primary outcome, the present study extends this literature, and notably, although no group-level differences were detected, approximately one-third of participants achieved clinically meaningful improvements,^39^ indicating individual-level benefits. Although the present study was not designed to identify predictors of treatment response, the findings suggest that some individuals derive meaningful benefit from exercise. Given the potential benefits and the limited treatment options available, an individualized exercise intervention may remain a reasonable treatment option for individuals with COVID-19–induced POTS, particularly as responders cannot yet be identified in advance.

The reduced upright time in the intervention group may reflect behavioral substitution or activity redistribution rather than reduced function. At follow-up, participants in the intervention group were likely performing supine exercise, and the observed reduction of approximately 30 minutes per day corresponded closely to the estimated prescribed exercise duration. This raises the possibility that a limited activity capacity was redistributed toward the intervention, replacing activities such as walking, to remain within the individual’s energy envelope^40^ rather than increasing overall daily activity. The concurrent reduction in daily steps is consistent with this interpretation and may indicate adherence to the intervention. However, these findings should be interpreted with caution, as exercise duration was not assessed and it therefore remains uncertain whether the intervention can account for the observed changes. The reductions in upright time and daily steps may also be explained by symptom fluctuations, PEM, behavioral adaptations, or reduced activity tolerance.^13^^,^^14^

The low-intensity nature of the intervention may have been insufficient to induce physiological adaptations beyond standard care. Previous POTS trials have typically applied moderate intensity, with substantial variability in total exercise dose,^6^ and suggest that higher exercise dose and adherence are associated with improvements in aerobic capacity,^37^ and similar patterns are suggested in PCC populations,^9^ supporting a dose-response relationship. The findings are unlikely to be explained solely by insufficient intervention exposure, as participants in the intervention group reported a median of 2.6 sessions per week and a mean exercise intensity within the light-to-moderate range. Nevertheless, the effectiveness of low-intensity exercise remains uncertain, particularly given the need to balance physiological adaptation, exercise tolerance and symptom exacerbation in this population.^8^^,^^13^^,^^14^ Within this context, the intervention appeared successful in supporting tolerability, and adherence, as reflected by lower attrition and intervention discontinuation than in previous exercise studies in PCC^12^ and POTS,^6^ alongside a tendency toward reduced PEM prevalence. The systematic evaluation of PEM is a key contribution of this study, as it has not previously been examined in exercise interventions for individuals with POTS^6^^,^^13^ or PCC.^8^ The potential PEM reduction should be interpreted cautiously in light of the wide credibility intervals, the lack of statistical significance, and the exploratory nature of the analysis. Taken together, low attrition and a trend toward reduced PEM prevalence suggest that this approach may facilitate sustained participation and enable progression toward higher cumulative exercise exposure, while minimizing the risk of symptom exacerbation. As such, this approach may provide a safe framework for introducing exercise in individuals with COVID-19–induced POTS.

Beyond PEM, the other secondary outcomes provided additional insight into functional, symptomatic, and mechanistic aspects of the intervention. Although no between-group differences were observed in the exploratory analyses, improvements over time in physical capacity, patient-identified activity limitations, and symptom burden suggested positive trajectories in both groups. These findings should also be interpreted with caution given the exploratory nature of the analyses, no correction for multiple testing, the large variability across outcomes, and possibly underpowered to detect between-group differences in the secondary outcomes. Nevertheless, the findings underscore the importance of recognizing COVID-19–induced POTS and ensuring access to standard care, as affected individuals may experience meaningful improvement over time.

Previous exercise studies in POTS have included few and heterogeneous PROMs,^10^^,^^37^^,^^38^^,^^41^ limiting comparability across the studies. By incorporating a broader range of PROMs, including multiple dimensions of fatigue, sleep, and mental health, the present study adds novel and clinically relevant information on these highly burdensome symptoms. These findings further highlight the uncertainty regarding exercise-specific effects on symptom burden and underscore the need for longer follow-up and targeted outcome measures to capture potential for differential responses over time.

The novel evaluation of muscle strength showed largely preserved strength at the group level with no changes over time, suggesting that peripheral muscle weakness is unlikely to be a primary driver of symptom burden or activity limitations in POTS. However, the substantial interindividual variability suggests potential relevance for resistance training in selected individuals, although its role in POTS populations has yet to be established.^6^

The generalizability of the findings should be interpreted in the context of the study population. The study included only individuals with COVID-19–induced POTS, recruited within routine Swedish clinical care approximately 2.6 years after the onset of the triggering COVID-19 infection. The predominance of women is consistent with the epidemiology of POTS and PCC.^1^, ^2^, ^3^, ^4^, ^5^ Consequently, the findings may not be generalizable to other POTS phenotypes, including those associated with other postviral syndromes, hypermobility spectrum disorders, or mast cell activation disorders. It cannot be excluded that exercise may be more effective in some of these prepandemic phenotypes, as previously demonstrated.^6^^,^^10^^,^^11^ The broad inclusion criteria and pragmatic intervention design enhance clinical relevance and support applicability to clinical practice. Generalizability to individuals at earlier or later stages of disease remains uncertain, as differences in symptom duration and access to care may influence treatment response.

### Study limitations

This study has several limitations and should be acknowledged when interpreting the findings. First, moderate-to-vigorous physical activity was not assessed, and reliance on step count and upright time only captures selected dimensions of physical activity without reflecting intensity or overall work load. Second, although the exercise logs enabled estimation of exercise frequency and perceived intensity, detailed dose-response analyses were not feasible because exercise modality, duration, and adherence to the individualized prescriptions were not systematically recorded. As a result, cumulative exercise dosage could not be quantified. In addition, the validity of the exercise log data cannot be verified, and it remains uncertain to what extent it reflects actual exercise behavior. The variability in the number of reported exercise sessions may reflect true differences in exercise performance, but may also indicate incomplete reporting. Given the importance of dose and adherence,^9^^,^^37^ these limitations may have attenuated the ability to detect intervention effects and to interpret the potential of dose-response relationships.

In addition, physical activity in the control group was unrestricted, allowing for engagement in self-selected activities throughout the study period. Furthermore, exercise logs were collected exclusively in the intervention group, precluding assessment of overall exercise exposure in the control group. Consequently, some participants in the control group may have achieved levels of physical activity comparable to the intervention group, thereby reducing the between-group differences. Although this approach enhances external validity, it limits causal attribution. Restricting usual care or physical activity in the control group was, however, not considered ethically justifiable.

The involvement of multiple assessors may have introduced measurement variability, particularly for muscle strength.^42^ In addition, PEM was analyzed as a binary outcome, which does not capture variation in severity or symptom burden; a more nuanced measure could have provided even deeper insight into PEM and its responsiveness to the intervention.

Despite these limitations, the randomized design and the combined use of patient-reported and objective outcomes, extending beyond hemodynamic measures, support a comprehensive evaluation of intervention effects.

## Conclusions

In this randomized trial, tailored exercise was not superior to standard care in improving HRQoL or upright time in COVID-19–induced POTS. Although no overall superiority was observed, the intervention was well tolerated and may still represent a reasonable therapeutic option for selected individuals with COVID-19–induced POTS. Finally, marked interindividual variability and heterogeneous recovery trajectories across outcomes highlight the need for exercise interventions to be individualized, carefully titrated, and integrated with pharmacologic and nonpharmacologic strategies. Future studies should aim to identify responders to exercise treatment, define the optimal exercise dose for clinical benefit, and include longitudinal follow-up to assess changes over time in this population.

Perspectives **COMPETENCY IN PATIENT CARE AND PROCEDURAL SKILLS:** Individuals with COVID-19-induced POTS may experience meaningful improvements over time when receiving comprehensive standard care. Although tailored exercise did not provide additional benefits at the group level, it was well tolerated and was associated with clinically meaningful improvements in some individuals, supporting its use as an individualized treatment option.

**TRANSLATIONAL OUTLOOK:** Further research is needed to identify predictors of treatment response to exercise in individuals with COVID-19-induced POTS. Improved understanding of patient characteristics associated with benefit may help guide personalized rehabilitation strategies and optimize clinical outcomes.

## Funding support and author disclosures

This study was supported by grants from the Swedish Heart Lung Foundation (20210024, 20210539, 20220563), Stockholm, Sweden; The 10.13039/501100004359Swedish Research Council (2021-02844), Stockholm, Sweden; The 10.13039/100018870Swedish Heart and Lung Association (FA 2022/32), Stockholm, Sweden. The study was also supported by Odd Fellows (B10), Stockholm, Sweden, and Stiftelsen Bygg-Göta Vetenskaplig forskning och Social hjälpverksamhet, Göteborg, Sweden. Dr Fedorowski reports consultancy and lecture fees from Medtronic Inc, Finapress Medical Systems, ArgenX, and Regeneron. All other authors have reported that they have no relationships relevant to the contents of this paper to disclose.
