# Supplementary material for: Effectiveness of Individualized Physical Exercise in COVID-19–Induced Postural Orthostatic Tachycardia Syndrome
Source: JACC Adv. 2026 Sep 22;5(11):103328. doi: 10.1016/j.jacadv.2026.103328 (PMC13685931; doi:10.1016/j.jacadv.2026.103328)
Supplement: Supplementary file 1 [file mmc1.docx]

# Supplemental Appendix

Table of Contents

[Supplemental Appendix	1](#_Toc239327198)

[**Supplemental Methods**	2](#_Toc239327199)

[**Supplemental Table 1**	12](#_Toc239327204)

[**Supplemental Table 2**	13](#_Toc239327205)

[**Supplemental Table 3**	14](#_Toc239327206)

[**Supplemental Table 4**	15](#_Toc239327207)

[**Supplemental Table 5**	16](#_Toc239327208)

[**Supplemental Material – CONSORT Checklist**	17](#_Toc239327209)

## **Supplemental Methods**

Information for individuals receiving treatment for POTS

Karolinska University Hospital

Physiotherapy

08-123 736 36

# **What is POTS?**

POTS, or Postural Orthostatic Tachycardia Syndrome, can be triggered, among other things, after a viral infection like COVID-19. POTS means that the body’s ability to regulate heart rate and blood pressure is impaired due to dysfunction in the autonomic nervous system. If you have been diagnosed with POTS, you may experience a range of symptoms, such as fatigue, exhaustion, heart palpitations, dizziness, shortness of breath, fainting, sleep difficulties, visual disturbances, chest discomfort, nausea, or problems with concentration. Symptoms often worsen when changing position to sitting or standing, or during physical activity.

# **Treatment** **Pharmacological treatment**

Symptom‑relieving medications that regulate heart rate or blood pressure may be used as part of the treatment for POTS. Other medications aimed at relieving symptoms may also be considered to help improve your well‑being. Your physician will determine whether medication is appropriate for you, and if so, which type and dosage are most suitable.

**Self‑care**
To reduce symptoms, you may consider the following:

- Avoid high temperatures/heat
- Avoid or reduce alcohol intake
- Drink plenty of fluids; your physician or dietitian will advise you on the amount that is appropriate for you. A common recommendation is 2–3 liters per day
- Increase your salt intake, for example by adding extra salt to your food; your physician or dietitian will advise what is appropriate for you.
- Eat smaller meals
- Use compression garments (if needed it can be prescribed and individually fitted by a healthcare provider)
- Avoid long periods of standing upright as well as lying completely flat for extended periods; for example, elevate the head of the bed and try to remain in a seated position during the day
- **Use strategies to reduce dizziness and fainting both before and during periods of standing, as described below.**

*Methods to reduce or prevent dizziness and fainting tendencies*

- Cross your legs
- Pump your feet and tighten your calf muscles
- Squeeze your gluteal muscles
- Sit down on a chair or squat down
- If symptoms become severe, lie down with your legs elevated, for example against a wall or supported by pillows
- When symptoms improve, stand up slowly and gradually; repeat the procedure if symptoms persist

## **Physical Exercise**

Adapted physical activity and exercise can be an important part of POTS treatment—both to reduce symptoms and to improve the condition over time. You will receive support from a physiotherapist to adjust the level and type of exercise you need, which often involves a gradual increase in training load. The exercise program should include both endurance and strength training, and treatment typically continues over several months.

**Aerobic training**

- Always begin with a gentle 5-10minute warm-up at a slow pace, and it should feel very easy (<9 on the Borg RPE scale)
- Then start the active training phase. This is adjusted with support from your physiotherapist, but usually begins in a lying position and with only a few minutes, which can be increased gradually as long as training does not lead to worsening symptoms.
- At the beginning, the exertion during training should feel light (11 in Borg RPE)
- Always finish with a cool-down at a slower pace for 5-10 minutes during which it should feel very easy (<9 in on the Borg RPE)

**Strength training**

- Follow your individualized program; start with few repetitions and few sets
- Take breaks whenever you need
- The goal is to complete 8-10 repetitions x 3 sets
- Self-rate your exertion during and after training using the Borg RPE scale. During training, the effort should feel light to moderately strenuous (11–13 on the Borg RPE scale)
- *Schedule 2 days/week where you take a break from training. On these days, you may rest, engage in other activities that you find enjoyable, or choose activities that are less physically demanding.*
- *Ideally, avoid having more than two consecutive days without training.*
- *Aim to complete at least four training sessions per week.*

**To make training easier**

Follow your prescribed medications and fluid intake recommendations

Avoid training in warm environments if possible

**Examples of strength exercises to perform in a gym setting**

**Examples of strength exercises to perform in a home setting**

## **Supplemental Table 1**

| **Supplementary Table 1 - Contrasts from LMM – EQ-VAS** | | | | | | | |
| --- | --- | --- | --- | --- | --- | --- | --- |
| **Type** | **Scope** | **Contrast** | **Estimate** | **SE** | **Lower 95% CI** | **Upper 95% CI** | **p-value** |
| Within-group | Control | 16 weeks - Baseline | 7.16 | 3.32 | 0.51 | 13.80 | **0.035** |
| Within-group | Intervention | 16 weeks - Baseline | 5.43 | 3.07 | -0.72 | 11.58 | 0.082 |
| Between-group | 16 weeks | Control - Intervention | 2.32 | 4.53 | -6.69 | 11.33 | 0.610 |
| Between-group | Baseline | Control - Intervention | 4.04 | 4.65 | -5.20 | 13.28 | 0.387 |
| Difference-in-differences | Overall |  | -1.73 | 4.52 | -10.78 | 7.33 | 0.704 |

## **Supplemental Table 2**

| **Supplemental Table 2 – Contrasts of linear mixed model for upright time (Minutes/day)** | | | | | | | |  |
| --- | --- | --- | --- | --- | --- | --- | --- | --- |
| **Type** | **Scope** | **Contrast** | **Estimate** | **SE** | **Lower 95% CI** | **Upper 95% CI** | **p-value** | |
| Within-group | Control | 16w – Baseline | 8.6 | 9.7 | -10.5 | 27.7 | 0.38 | |
| Within-group | Intervention | 16w – Baseline | -22.7 | 8.6 | -39.7 | -5.8 | **0.01** | |
| Between-group | 16 weeks | Control – Intervention | 22.8 | 30.4 | -38.1 | 83.7 | 0.46 | |
| Between-group | Baseline | Control – Intervention | -8.5 | 30.2 | -69.0 | 52.0 | 0.78 | |
| Difference-in-differences | Overall | (16w − Baseline) Intervention – (16w − Baseline) Control | -31.3 | 13.0 | -56.8 | -5.8 | **0.02** | |

## **Supplemental Table 3**

| **Supplemental Table 3 – Contrasts of linear mixed model for MaPS** | | | | | | | |
| --- | --- | --- | --- | --- | --- | --- | --- |
| **Type** | **Scope** | **Contrast** | **Estimate** | **SE** | **Lower 95% CI** | **Upper 95% CI** | **p-value** |
| Within-group | Control | 16 weeks - Baseline | -5.47 | 2.73 | -10.95 | 0.01 | **0.050** |
| Within-group | Intervention | 16 weeks - Baseline | -4.50 | 2.48 | -9.48 | 0.49 | 0.076 |
| Between-group | 16 weeks | Control - Intervention | -3.15 | 5.34 | -13.81 | 7.51 | 0.558 |
| Between-group | Baseline | Control - Intervention | -4.12 | 5.43 | -14.95 | 6.71 | 0.450 |
| Difference-in-differences | Overall |  | 0.97 | 3.69 | -6.43 | 8.38 | 0.793 |

## **Supplemental Table 4**

| **Supplemental Table 4 – Contrasts from linear mixed model for % of predicted 6MWT** | | | | | | | |
| --- | --- | --- | --- | --- | --- | --- | --- |
| **Type** | **Scope** | **Contrast** | **Estimate** | **SE** | **Lower 95% CI** | **Upper 95% CI** | **p-value** |
| Within-group | Control | 16 weeks - Baseline | 4.64 | 1.86 | 0.91 | 8.36 | **0.016** |
| Within-group | Intervention | 16 weeks - Baseline | 4.26 | 1.66 | 0.93 | 7.59 | **0.013** |
| Between-group | 16 weeks | Control - Intervention | 3.70 | 4.80 | -5.88 | 13.29 | 0.443 |
| Between-group | Baseline | Control - Intervention | 4.08 | 4.88 | -5.65 | 13.82 | 0.405 |
| Difference-in-differences | Overall |  | -0.38 | 2.49 | -5.38 | 4.62 | 0.880 |

## **Supplemental Table 5**

| **Supplemental Table 5 – Contrasts from linear mixed model for PSFS** | | | | | | | |
| --- | --- | --- | --- | --- | --- | --- | --- |
| **Type** | **Scope** | **Contrast** | **Estimate** | **SE** | **Lower 95% CI** | **Upper 95% CI** | **p-value** |
| Within-group | Control | 16 weeks - Baseline | 0.87 | 0.24 | 0.38 | 1.36 | **0.001** |
| Within-group | Intervention | 16 weeks - Baseline | 0.90 | 0.22 | 0.46 | 1.35 | **0.000** |
| Between-group | 16 weeks | Control - Intervention | 0.36 | 0.42 | -0.47 | 1.19 | 0.391 |
| Between-group | Baseline | Control - Intervention | 0.33 | 0.43 | -0.53 | 1.18 | 0.446 |
| Difference-in-differences | Overall |  | 0.03 | 0.33 | -0.63 | 0.69 | 0.927 |

## **Supplemental Material – CONSORT Checklist**

| **Section / Topic** | **No** | **CONSORT 2025 checklist item description** | **Reported on page no.** |
| --- | --- | --- | --- |
| **Title and abstract** | | |  |
| Title and structured abstract | 1a | Identification as a randomised trial | 3 |
|  | 1b | Structured summary of the trial design, methods, results, and conclusions | 3 |
| **Open science** | | |  |
| Trial registration | 2 | Name of trial registry, identifying number (with URL) and date of registration | 6 |
| Protocol and statistical analysis plan | 3 | Where the trial protocol and statistical analysis plan can be accessed | 6, 10 |
| Data sharing | 4 | Where and how the individual de-identified participant data (including data dictionary), statistical code and any other materials can be accessed | 11-12 |
| Funding and conflicts of interest | 5a | Sources of funding and other support (e.g., supply of drugs), and role of funders in the design, conduct, analysis and reporting of the trial | 1 |
|  | 5b | Financial and other conflicts of interest of the manuscript authors | 1 |
| **Introduction** | | |  |
| Background and rationale | 6 | Scientific background and rationale | 5-6 |
| Objectives | 7 | Specific objectives related to benefits and harms | 5-6 |
| **Methods** | | |  |
| Patient and public involvement | 8 | Details of patient or public involvement in the design, conduct and reporting of the trial | *Not Applicable* |
| Trial design | 9 | Description of trial design including type of trial (e.g., parallel group, crossover), allocation ratio, and framework (e.g., superiority, equivalence, non-inferiority, exploratory) | 6 |
| Changes to trial protocol | 10 | Important changes to the trial after it commenced including any outcomes or analyses that were not prespecified, with reason | 11 |
| Trial setting | 11 | Settings (e.g., community, hospital) and locations (e.g., countries, sites) where the trial was conducted | 6, 8 |
| Eligibility criteria | 12a | Eligibility criteria for participants | 6 |
|  | 12b | If applicable, eligibility criteria for sites and for individuals delivering the interventions (e.g., surgeons, physiotherapists) | 8 |
| Intervention and comparator | 13 | Intervention and comparator with sufficient details to allow replication. If relevant, where additional materials describing the intervention and comparator (e.g., intervention manual) can be accessed | 7, Supplementary |
| Outcomes | 14 | Pre-specified primary and secondary outcomes, including the specific measurement variable (e.g., systolic blood pressure), analysis metric (e.g., change from baseline, final value, time to event), method of aggregation (e.g., median, proportion), and time point for each outcome | 8-10 |
| Harms | 15 | How harms were defined and assessed (e.g., systematically, non-systematically) | *Not Applicable* |
| Sample size | 16a | How sample size was determined, including all assumptions supporting the sample size calculation | 10 |
|  | 16b | Explanation of any interim analyses and stopping guidelines | 10 |
| Randomisation: |  |  |  |
| Sequence generation | 17a | Who generated the random allocation sequence and the method used | 6 |
|  | 17b | Type of randomisation and details of any restriction (e.g., stratification, blocking and block size) | 6 |
| Allocation concealment mechanism | 18 | Mechanism used to implement the random allocation sequence (e.g., central computer/telephone; sequentially numbered, opaque, sealed containers), describing any steps to conceal the sequence until interventions were assigned | 6 |
| Implementation | 19 | Whether the personnel who enrolled and those who assigned participants to the interventions had access to the random allocation sequence | 6-7 |
| Blinding | 20a | Who was blinded after assignment to interventions (e.g., participants, care providers, outcome assessors, data analysts) | 6-7 |
|  | 20b | If blinded, how blinding was achieved and description of the similarity of interventions | 7 |
| Statistical methods | 21a | Statistical methods used to compare groups for primary and secondary outcomes, including harms | 11 |
|  | 21b | Definition of who is included in each analysis (e.g., all randomised participants), and in which group | 11, 28-29, Fig 2-5 |
|  | 21c | How missing data were handled in the analysis | 11 |
|  | 21d | Methods for any additional analyses (e.g., subgroup and sensitivity analyses), distinguishing prespecified from post-hoc | 11 |
| **Results** | | |  |
| Participant flow, including flow diagram | 22a | For each group, the numbers of participants who were randomly assigned, received intended intervention, and were analysed for the primary outcome | 12, Fig 2 |
|  | 22b | For each group, losses and exclusions after randomisation, together with reasons | 12, Fig 2 |
| Recruitment | 23a | Dates defining the periods of recruitment and follow-up for outcomes of benefits and harms | 12 |
|  | 23b | If relevant, why the trial ended or was stopped |  |
| Intervention and comparator delivery | 24a | Intervention and comparator as they were actually administered (e.g., where appropriate, who delivered the intervention/comparator, how participants adhered, whether they were delivered as intended [fidelity]) | 12, Fig 2 |
|  | 24b | Concomitant care received during the trial for each group | 28 |
| Baseline data | 25 | A table showing baseline demographic and clinical characteristics for each group | 28, 29 |
| Numbers analysed,  outcomes and estimation | 26 | For each primary and secondary outcome, by group:   - the number of participants included in the analysis - the number of participants with available data at the outcome time point - result for each group, and the estimated effect size and its precision (such as 95% confidence interval) - for binary outcomes, presentation of both absolute and relative effect size | 11, 28-29, Fig 3-5 |
| Harms | 27 | All harms or unintended events in each group | *Not Applicable* |
| Ancillary analyses | 28 | Any other analyses performed, including subgroup and sensitivity analyses, distinguishing pre-specified from post-hoc | 11 |
| **Discussion** | | |  |
| Interpretation | 29 | Interpretation consistent with results, balancing benefits and harms, and considering other relevant evidence | 14-18 |
| Limitations | 30 | Trial limitations, addressing sources of potential bias, imprecision, generalisability, and, if relevant, multiplicity of analyses | 18-19 |
